# Low-level voluntary input enhances corticospinal excitability during ankle dorsiflexion neuromuscular electrical stimulation in healthy young adults

**DOI:** 10.1371/journal.pone.0282671

**Published:** 2023-03-08

**Authors:** Akiko Yamaguchi, Atsushi Sasaki, Milos R. Popovic, Matija Milosevic, Kimitaka Nakazawa

**Affiliations:** 1 Department of Life Sciences, Graduate School of Arts and Sciences, The University of Tokyo, Meguroku, Tokyo, Japan; 2 Department of Rehabilitation Medicine I, Fujita Health University School of Medicine, Toyoake, Aichi, Japan; 3 Japan Society for the Promotion of Science, Chiyodaku, Tokyo, Japan; 4 Graduate School of Engineering Science, Osaka University, Toyonaka, Osaka, Japan; 5 Institute of Biomedical Engineering, University of Toronto, Toronto, Ontario, Canada; 6 KITE Research Institute, Toronto Rehabilitation Institute—University Health Network, Toronto, Ontario, Canada; 7 CRANIA, University Health Network & University of Toronto, Toronto, Ontario, Canada; BG-Universitatsklinikum Bergmannsheil, Ruhr-Universitat Bochum, GERMANY

## Abstract

Previous evidence indicated that interventions with combined neuromuscular electrical stimulation (NMES) and voluntary muscle contractions could have superior effects on corticospinal excitability when the produced total force is larger than each single intervention. However, it is unclear whether the superior effects exist when the produced force is matched between the interventions. Ten able-bodied individuals performed three intervention sessions on separate days: (i) NMES–tibialis anterior (TA) stimulation; (ii) NMES+VOL–TA stimulation combined with voluntary ankle dorsiflexion; (iii) VOL–voluntary ankle dorsiflexion. Each intervention was exerted at the same total output of 20% of maximal force and applied intermittently (5 s ON / 19 s OFF) for 16 min. Motor evoked potentials (MEP) of the right TA and soleus muscles and maximum motor response (M_max_) of the common peroneal nerve were assessed: before, during, and for 30 min after each intervention. Additionally, the ankle dorsiflexion force-matching task was evaluated before and after each intervention. Consequently, the TA MEP/M_max_ during NMES+VOL and VOL sessions were significantly facilitated immediately after the interventions started until the interventions were over. Compared to NMES, larger facilitation was observed during NMES+VOL and VOL sessions, but no difference was found between them. Motor control was not affected by any interventions. Although superior combined effects were not shown compared to voluntary contractions alone, low-level voluntary contractions combined with NMES resulted in facilitated corticospinal excitability compared to NMES alone. This suggests that the voluntary drive could improve the effects of NMES even during low-level contractions, even if motor control is not affected.

## Introduction

Neuromuscular electrical stimulation (NMES) is an effective technique for generating muscle contractions in rehabilitation for people with neurological impairments [1–3]. Previous studies have shown that NMES can affect the excitability of both spinal and supraspinal circuits [4–7]. It has also been demonstrated that neural effects of short-term application of NMES can persist for at least 30 min after the intervention, which suggests that NMES of muscles or peripheral nerves can affect the central nervous system neuroplasticity even after the stimulating period to possibly improve motor control [4, 5, 7–9].

Active (voluntary) engagement is an important factor in rehabilitation after neurological impairments [10]. It is well known that voluntary motor commands from the motor cortex during NMES can contribute to neuromodulation of the spinal reflex circuits [11–13] and supraspinal circuits [2, 14–16]. Possible mechanisms of increased corticospinal excitability during NMES combined with voluntary contractions have been explained by the Hebbian plasticity [17] like-synaptic strengthening [4, 7, 16, 18, 19] and unmasking of silent synaptic connections [20, 21] due to afferent inputs, which are electrically evoked afferent volley and reafferent inputs of sensory signals, and efferent outputs generated by simultaneous voluntary muscle contractions within the spinal cord and motor cortex. To date, it has been reported that combining NMES with voluntary movements can enhance and retain corticospinal excitability to a larger extent compared to NMES alone or voluntary muscle contractions alone [5, 7, 23]. The previous studies [5, 7, 23] applied NMES additionally during voluntary contractions, which produced a larger total force output compared to each single intervention as the consequence. Producing the larger total force may have enhanced corticospinal excitability to activate motoneurons by efferent central commands (i.e., descending voluntary drive) and stronger afferent volleys produced by NMES [24]. Since previous studies have shown that voluntary muscle contraction alone [22, 23] as well as NMES alone [21, 24–26] can increase corticospinal excitability, the combined effect of NMES and voluntary muscle contractions may produce additive effects. Overall, this raised a question of whether superior corticospinal excitability modulation effects could also be observed even when the intensities of NMES and voluntary muscle contractions were lower than each single intervention under the condition of matched total force output between the interventions. If the superior effects could still be observed, we could conclude that the combined effects were induced by synaptic strengthening in the spinal and/or cortical motor circuits via pairing effects, rather than the additive effects of greater total force output. It is assumed that repetitive presynaptic activation induced by afferent inputs from NMES may enhance synaptic strengthening [4, 16, 18] and unmasking of silent connections [20, 21] within the cortical circuits, resulting in firing of postsynaptic neurons. There is also a possibility that increased activity between pre- and postsynaptic neurons may occur within the spinal cord due to the afferent inputs from NMES and descending voluntary drive [19, 27]. Furthermore, it is known that sufficient NMES intensity plays a key role for increasing corticospinal excitability [28], while a previous study showed that combined intervention of voluntary muscle contractions with low-level NMES induced greater corticospinal facilitation than that with high-level NMES [29]. Thus, it can also be suggested that low-level NMES might also have effects on the facilitation of corticospinal excitability when combined with voluntary muscle contractions. However, it is unknown whether such pairing effects are induced when the low-level interventions are combined. Hence, clarifying these questions can provide insights into the underlying mechanisms of NMES neuromodulation effects.

Furthermore, NMES can contribute to improving motor control [18, 30]. Evidence shows that acquiring motor skills is associated with increased corticospinal facilitation during motor learning [31, 32]. However, only a handful of studies have investigated the association between short-term effects of NMES on corticospinal excitability and motor control [33, 34]. McDonnell and Ridding [33] demonstrated that NMES before finger motor training induced facilitation of motor cortical excitability and improvement of speed and accuracy of fine motor control more rapidly compared to the group that did not receive NMES, while Summers et al. [34] showed that pre-conditioning NMES prior to visuo-motor adaptation tasks resulted in faster motor learning (i.e., improved adaptation rate) along with an increase in corticospinal excitability, but without any significant differences in motor control performance changes (i.e., speed and accuracy) between NMES and sham groups. The application of NMES on muscles or nerves activates the peripheral motor axons directly, besides evoking the sensory volleys that activate synaptic transmission from Ia afferents to motoneurons in the spinal cord and indirectly generating sustained motoneuron discharge [27, 35, 36]. Therefore, it is assumed that such motoneuron activation may induce quicker response times with less error in the ballistic contractions which discharge rate preferentially involves [37], along with increased corticospinal excitability. In addition, neuroimaging studies have reported that NMES activates the somatosensory areas which are responsible for sensory information processing and sensorimotor integration [38–40]. Thus, NMES might possibly contribute to better motor control. A previous study in stroke patients demonstrated that NMES on the wrist extensor muscles for 10 weeks induced greater improvements in accuracy of finger movement and the onset/offset of muscle contractions accompanied by increased cortical activation in the contralateral motor cortex compared to non-stimulated therapy [41]. However, it remains unclear whether short-term stimulation can immediately alter motor control performance, especially in speed and accuracy.

Therefore, the aim of the current study was to investigate the effects of NMES and voluntary ankle dorsiflexion contractions on corticospinal excitability when the total generated force was matched between the following interventions: (i) NMES alone, (ii) NMES combined with voluntary contractions, and (iii) voluntary contractions alone. In addition, the effects on motor control were investigated before and after each intervention. We hypothesized that the combined intervention would facilitate corticospinal excitability to a greater extent compared to each intervention alone [5] despite matched force output between the interventions. Moreover, we hypothesized that NMES interventions would affect motor control (i.e., increase speed and accuracy) [33, 41] due to increased excitation of the motoneuron pool [27] even after short-duration interventions.

## Materials and methods

### Participants

Ten able-bodied individuals (eight males and two females; 25.0±1.7 years) participated in this study. All participants were right foot dominant determined using a questionnaire [42]. None of the participants had a history of neuromuscular and other injuries and any contraindications for TMS experiments [43]. Participants were asked to refrain from consuming alcohol 24 h prior to the experiments. All participants provided written informed consent in accordance with the principles of the Declaration of Helsinki prior to joining the study. The study protocol was approved by the local ethics committee of the Graduate School of Arts and Sciences at The University of Tokyo.

### Experimental setup

**Fig 1:**
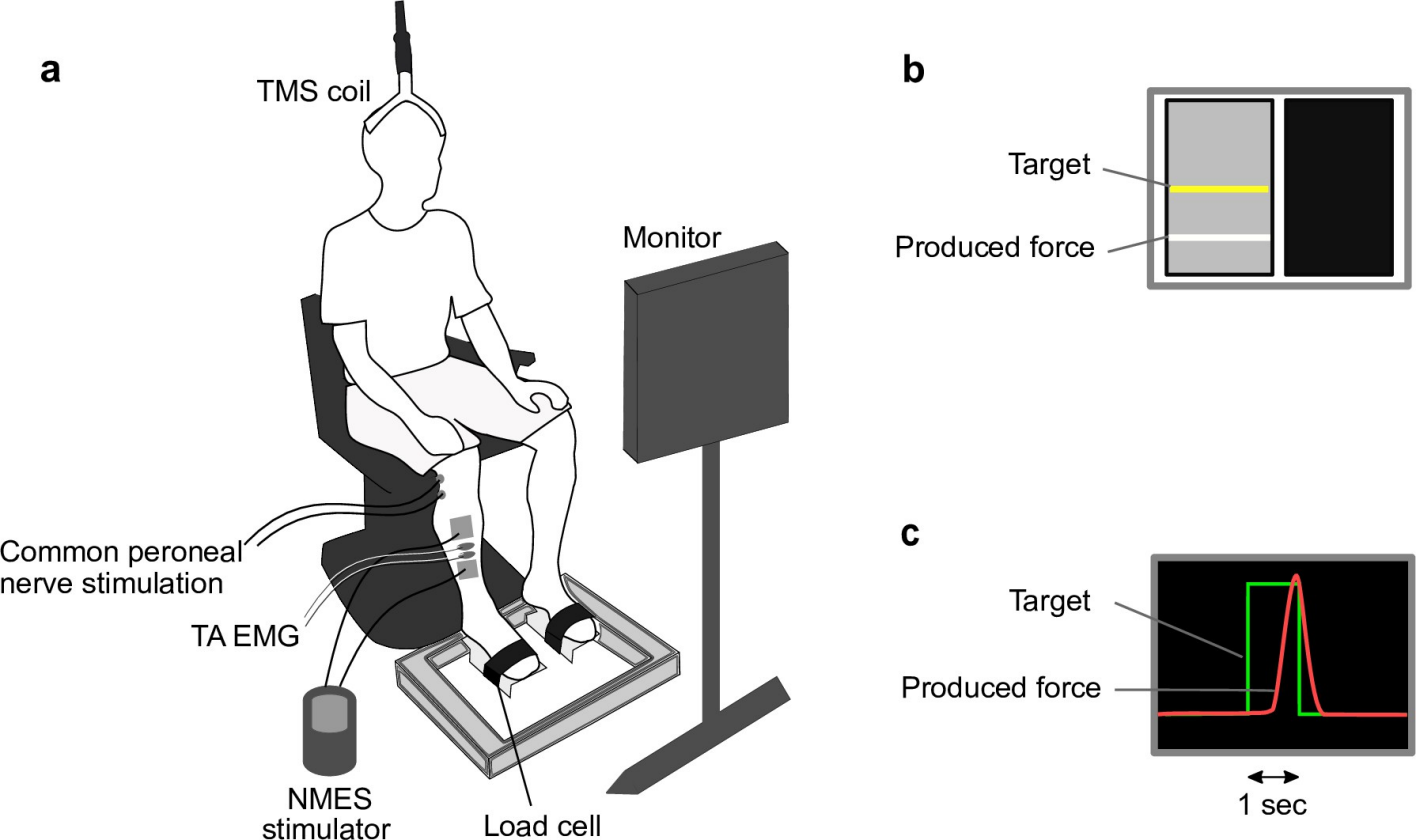
Experimental setup. **(a)** NMES was applied on the right TA during ON period (5 s). During OFF period (19 s), TMS and peripheral nerve stimulation were applied; **(b)** Participants were instructed to match the ankle dorsiflexor force to a target when the panel changed to the ON signal (black screen). The yellow line represented the target. Visual force feedback was presented using a white line; and **(c)** Force-matching task consisted of right leg isometric ankle dorsiflexion during 1 s contractions. The green line represented the target. Visual force feedback was presented using a red line. Abbreviations: EMG, electromyography; NMES, neuromuscular electrical stimulation; TA, tibialis anterior; TMS, transcranial magnetic stimulation.

The experimental setup is shown in Fig 1A. Participants were seated on a height adjustable chair with their hips, knees, and ankles flexed approximately at 90°. Each foot was securely strapped to a metal footplate, which was attached to a strain-gauge load cell (LCB03K060L, A&D Inc., Tokyo, Japan) [44]. During muscle contractions, participants were asked to maintain their posture and to try to avoid hip flexor and knee extensor contractions, while the experimenters carefully observed their posture and movements throughout the interventions. A computer monitor was placed in front of the participants at a distance of 70 cm at their eye-level (Fig 1A).

Prior to starting each intervention of the experiment, participants performed ankle dorsiflexor isometric maximum voluntary contraction (MVC) to record maximal force exertion. After a warm-up, participants were instructed to maximally flex their right ankle for 3 s in the isometric contraction condition. Two repeated trials were performed with a rest period of at least 1 min between trials [45]. During the MVC trials, participants were verbally encouraged to produce a maximal force exertion. The average force in the middle 2-s windows was obtained and averaged between the two repeated trials to determine the MVC force level for each participant.

### Experimental protocol

**Fig 2:**
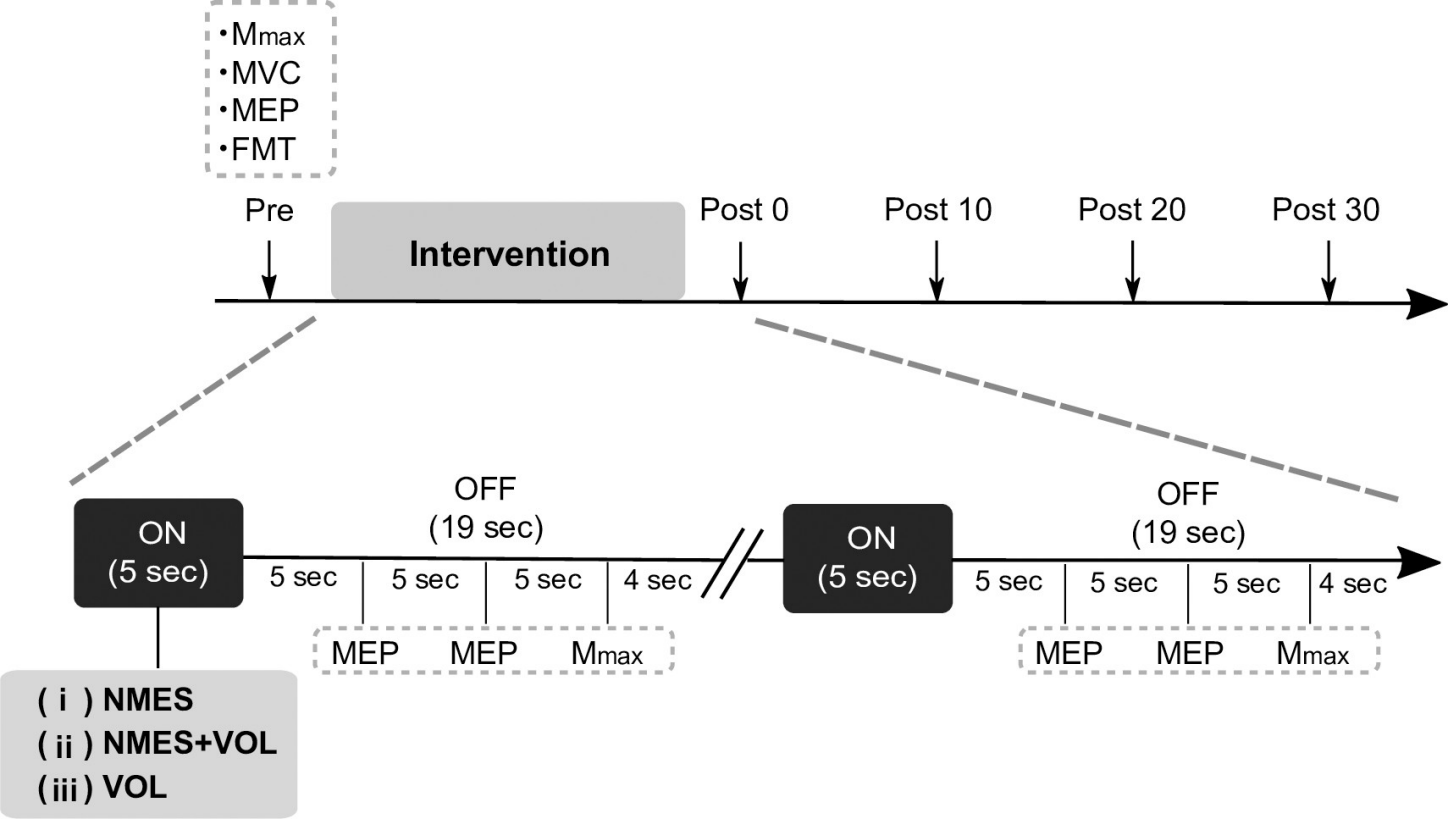
Time course of the experimental protocol. Participants performed three interventions: (i) NMES alone; (ii) NMES+VOL; and (iii) VOL alone. The assessments of MEP and M_max_ were measured before starting the interventions (Pre), during the interventions in the NMES OFF periods (During1 (0–4 min), During2 (4–8 min), During3 (8–12 min), During4 (12–16 min)), as well as immediately (Post0), 10 min (Post10), 20 min (Post20), and 30 min (Post30) after the interventions. Abbreviations: FMT, force-matching task; MEP, motor evoked potential; MVC, maximum voluntary contraction; NMES, neuromuscular electrical stimulation; VOL, voluntary contractions.

All participants performed three intervention sessions: (i) NMES–right leg TA muscle was activated using NMES at an intensity that produced 20% of isometric MVC force; (ii) NMES+VOL–participants maintained voluntary right ankle dorsiflexion at 10% of MVC force, while NMES increased the total force output to 20% of MVC force; and (iii) VOL–participants maintained voluntary right ankle dorsiflexion at 20% of MVC force. Each intervention was applied on separate days with at least one day between interventions and the order of interventions was randomized among participants. All interventions were applied intermittently: 5 s ON / 19 s OFF, for a total of 40 cycles, a total 16-min period [(5+19) s × 40 cycles = 960 s / 16 min]. During the interventions, the target force level was set at 20% of MVC force and the real-time visual feedback of the current force level was displayed on the left side of the computer monitor with a yellow and a white bar, respectively (Fig 1B). The visual feedback of the force was provided using a custom-made application in LabVIEW (National Instruments Corporation, Austin, TX, USA). On the right side of the computer monitor, a visual cue displayed the ON period (screen was dark) and the OFF period (monitor displayed ‘relax’) to indicate when participants should perform ankle dorsiflexion and when to relax their muscles (Fig 1B). The visual cue for the performance timing was provided using a custom-made Matlab (The MathWorks, Natick, MA, USA) script. A data acquisition system (USB-6259 BNC, National Instruments, Austin, TX, USA) was used to record ankle force data at a sampling frequency of 4,000 Hz. During the interventions that required voluntary contractions (NMES+VOL and VOL), participants were instructed to match the current force level to the target line as quickly and precisely as possible when the ON signal appeared and to maintain the contraction until the computer monitor indicated the OFF period. Although voluntary engagement was not required during the NMES intervention, participants were asked to pay attention to the monitor to minimize the influence of visual attention. Assessments were performed in the following intervals: (i) before starting the interventions (Pre); (ii) during the interventions in each 19-s OFF periods (During); and (iii) immediately after (Post0), 10 min after (Post10), 20 min after (Post20), and 30 min after (Post30) the interventions (Fig 2). A custom Matlab (The MathWorks, Natick, MA, USA) script was used to trigger NMES through an Arduino microcontroller during the interventions as well as to synchronize assessment timing.

### Neuromuscular electrical stimulation (NMES)

For the NMES and NMES+VOL interventions, electrical stimulation was applied over the right TA muscle (Fig 1A). A portable constant-current electrical stimulator (Complex Motion, Compex, Switzerland) was used to deliver NMES by applying a rectangular, biphasic, asymmetric charge-balanced stimulation pulses with a 300 μs pulse width at a frequency of 40 Hz via surface electrodes (anode: 5×5 cm, cathode: 5×5 cm) [1, 13]. During setup prior to the experiments, the placement of electrodes was first determined by placing the anode on the nerve end and the cathode on the muscle belly. The cathode was adjusted such that it was positioned over the motor point and corrected if necessary to produce smooth muscle contractions (i.e., no inversion or eversion). During the NMES intervention, the stimulation amplitude was increased and determined to produce 20% of MVC force at the first cycle of the intervention. Then, from the second cycle, NMES was delivered simultaneously when the ON signal appeared, while participants remained at rest. During the NMES+VOL intervention, the stimulation intensity was increased immediately after the voluntarily produced force reached 10% MVC force and was determined to produce a total of 20% MVC force output at the first cycle of the intervention. From the second cycle, the NMES was delivered when the ON signal appeared (indicating to produce voluntary muscle contractions). The stimulation amplitudes ranged between 18 and 40 mA with final a setting of 31.4±5.0 mA (mean±SD) for the NMES intervention to produce 20% of MVC force, which was significantly higher than that of 26.5±6.7 mA for the NMES+VOL intervention to provide an additional force during voluntary isometric ankle dorsiflexion and a total output of 20% MVC force (*t*(9) = 3.07, *p* = .013).

### Electromyography

Electromyography (EMG) signals were obtained from the right TA and soleus muscles using bipolar Ag–AgCl surface electrodes (Vitrode F-150S, Nihon Koden, Tokyo, Japan). The recording EMG electrodes were placed over the right TA muscle between the anode and the cathode of the NMES electrodes [46] as illustrated in Fig 1A. The recording electrodes of the soleus muscle were placed on the muscle belly. These electrodes were placed on each muscle with approximately 2 cm separation. The EMG signals were amplified (×1,000) and band-pass filtered at 5–1,000 Hz using a multichannel EMG amplifier (MEG-6108, Nihon Koden, Tokyo, Japan). All EMG data were digitized using an analog-to-digital converter at a sampling frequency of 4,000 Hz (PowerLab 16/s, AD Instruments, NSW, Australia) and saved on the computer for post-processing. The baseline background EMG was determined 50 ms prior to TMS stimulation.

### Motor evoked potentials

Motor evoked potentials (MEP) in the right TA and soleus muscles were elicited using single-pulse TMS applied over the contralateral (left) primary motor cortex using a magnetic stimulator (Magstim 200^2^, Magstim Company, Whitland, UK) through a double-cone coil. The coil was placed to induce the current flow in the posterior-anterior direction in the brain [47]. The optimal stimulation site for the TA (“hot spot”) was searched at the area that evoked the largest responses, typically at the location 1 cm lateral and 1 cm posterior to the vertex [48], and the location was tracked using a neuronavigation system (Brainsight, Rogue Research, Montreal, Canada). Resting motor threshold (rMT) was determined as the lowest intensity that evoked a peak-to-peak MEP amplitude greater than 50 μV in at least five out of ten successive trials [43]. The stimulation intensity was then set to 120% of rMT to evoke responses in the TA and soleus muscles simultaneously at rest. The hot spot and stimulation intensity were determined prior to each intervention. There were no statistically significant differences in the stimulation intensity between the interventions [NMES: 61.3±9.6% of maximum stimulator output (MSO); NMES+VOL: 61.1±8.6% MSO; VOL: 62.9±11.2% MSO; *F*_2,18_ = .920, *p* = .417]. For the Pre and Post assessments, ten stimuli were delivered, and their responses were averaged. For the During assessment, two stimuli were delivered in each 19-s OFF period at an inter-stimulus interval of 5 s (Fig 2). The total intervention duration was divided into 4 windows: During1 (0–4 min), During2 (4–8 min), During3 (8–12 min), and During4 (12–16 min). Consequently, twenty MEP responses in each time window were used for the analysis. Note that different time windows were also tested, and the results were consistent, while we only report the 4-min time window results herein.

### Maximum motor response

Maximum motor response (M_max_) was elicited by stimulating the common peroneal nerve using an electrical stimulator (DS7A, Digitimer Ltd., Hertfordshire, UK) to apply a single monophasic square pulse with a 1 ms pulse duration via surface electrodes. Specifically, the cathode was placed below the head of the fibula and the anode was placed approximately 2 cm distally to the cathode as shown in Fig 1A [9]. The stimulus intensity was increased until reaching the response plateau. To obtain the M_max_, the stimulation current was set to 120% of that intensity and kept constant for the duration of the experiment [49]. For the Pre and Post assessments, five stimuli were delivered, and their responses were averaged. For the During assessments, one stimulus was delivered in each OFF period, then ten responses were averaged in each During assessment (i.e., During1, During2, During3, and During4).

### Force-matching task

To investigate the effects of each intervention on motor control, an isometric ankle dorsiflexor force-matching task was tested before (i.e., Pre) and after each intervention (i.e., Pre, Post0, Post10, Post20, and Post30). The target for the force-matching task consisted of a square wave with a 1-s window that moved from right to left on the monitor every 3 to 5 s in random intervals (Fig 1C) as previously described [44]. The target level during the force-matching task was always set at 10% of the MVC force, which was relatively low to avoid muscle fatigue. It was suspected that if the NMES additionally affects the motoneuron pool at the spinal cord level, the firing pattern of motor units will be altered and the motor performance will change, even though the produced force level is low. Participants were instructed to match the force to the target as quickly and precisely as possible by performing ankle dorsiflexion when the rising edge of the target appeared and to relax immediately after reaching the target. Prior to the experiment, participants were given a practice period to become familiar with the task.

### Data analysis

Peak-to-peak amplitude of MEP responses for both the TA and soleus muscles were computed for all experimental conditions, for each trial, and each participant. Prior to computing MEP responses, background EMG activity in a 50 ms window before each TMS stimulus was defined by calculating the root mean square EMG activity in each trial. Trials were removed from further analysis if the background EMG activity exceeded three standard deviations of the baseline background EMG. In total, 1.8% of all trials were excluded before analysis due to baseline background EMG. Moreover, the peak-to-peak amplitudes of M_max_ responses from the TA muscle were also computed for each trial and each subject. The MEP peak-to-peak amplitudes of the TA were normalized to the M_max_ (MEP/M_max_) [50]. The TA MEP obtained at Pre and Post was normalized by the M_max_ obtained at Pre and Post, respectively. During Pre and Post assessment, we obtained ten MEPs and five M_max_. We normalized with one M_max_ for each of the two MEPs, then ten MEP/M_max_ values were averaged and used for the analysis. Regarding the During assessment, we obtained two MEPs and one M_max_ during the OFF period (Fig 2). We normalized with the one M_max_ for the two MEPs obtained during the same OFF period, then, twenty MEP/M_max_ values were averaged in 4-min time window and used for the analysis.

Force signals were first low-pass filtered at a frequency of 50 Hz. Using the filtered signals, the following outcome measures were computed to evaluate performance on the force-matching tasks: (i) error–difference between the percentage of the peak force and the target level (absolute error); and (ii) reaction time–time interval from the target appearance to the force onset, which was detected at the points where the force exceeded the mean plus three standard deviations of the baseline. All MEP, M_max_, and force-matching analyses were performed using a custom script in the Matlab (The MathWorks, Natick, MA, USA) and confirmed visually for each trial.

### Statistics

First, to verify the validity of the crossover measurement, comparisons of each outcome variable at the Pre time point between the interventions were performed using the Kruskal-Wallis test. Since the Shapiro-Wilk test showed that data were not normally distributed, the non-parametric test was used. Next, the intervention effects were compared. Since the Shapiro-Wilk test showed that the data were not normally distributed, log-transformation was applied prior to performing the analysis [16]. To determine intervention and time effects, the mixed-effects models [51] were used for dependent variables (background EMG, TA MEP/M_max_, soleus MEP, and M_max_). We included participants as a random effect in the models. Intervention (NMES, NMES+VOL, and VOL), time (During1, During2, During3, During4, Post0, Post10, Post20, and Post30), and intervention*time interaction were modeled as fixed effects. Pre values were modeled as the potential covariate_._ Model-based post hoc pairwise comparisons of estimated fixed effects of intervention with Bonferroni correction were conducted. The same analysis was used for motor control assessments (i.e., error and reaction time). Furthermore, since the primary focus of our study was changes in the corticospinal excitability and motor control performance, we applied Dunnett’s multiple comparison tests on the TA MEP/M_max_, soleus MEP, and motor control assessments to compare Pre and other time points for each intervention separately. All tests except for the calculation of effect size were performed using SPSS software package (IBM Inc., Armonk, New York, USA). The effect size was calculated using R version 4.2.1 and the effect size package. Statistical significance was set at *p* < .05.

## Results

### Assessments of values at the Pre time point

There were no significant differences between interventions in any outcome variables at the Pre time point (Table 1), suggesting validity of measurements across interventions.

**Table 1:** Outcome variables at the Pre time point and results of the statistical comparison between the interventions.

	Mean (SD)			*P*-value
	NMES	NMES+VOL	VOL	
MVC force (N)	363.5 (83.2)	361.5 (85.6)	373.04 (94.6)	.863
TA MEP/M_max_	0.07 (0.06)	0.06 (0.05)	0.09 (0.11)	.826
Soleus MEP (mV)	0.06 (0.04)	0.05 (0.02)	0.05 (0.02)	.867
M_max_ (mV)	3.41 (0.79)	4.46 (1.94)	3.81 (1.09)	.460
Error	1.63 (0.64)	1.86 (0.55)	2.11 (1.07)	.625
Reaction time (s)	0.47 (0.08)	0.46 (0.84)	0.42 (0.04)	.149

Abbreviations: MEP, motor evoked potentials; MVC, maximum voluntary contraction; NMES, neuromuscular electrical stimulation; TA, tibialis anterior; VOL, voluntary contractions.

### Background EMG activity

There were no significant main effects of intervention (*F*_2,206_ *=* .46, *p* = .629) and time (*F*_7,206_ *=* 1.83, *p* = .083) factors, as well as no significant interaction (*F*_14,206_ *=* 1.03, *p* = .424) for the background EMG activity of the TA muscle. Similarly, there were no significant main effects of intervention (*F*_2,206_ *=* .60, *p* = .548) and time (*F*_7,205_ *=* 1.95, *p* = .064) factors, as well as no significant interaction (*F*_14,205_ *=* .92, *p* = .539) for the background EMG activity of the soleus muscle.

### TA MEP/Mmax

**Fig 3:**
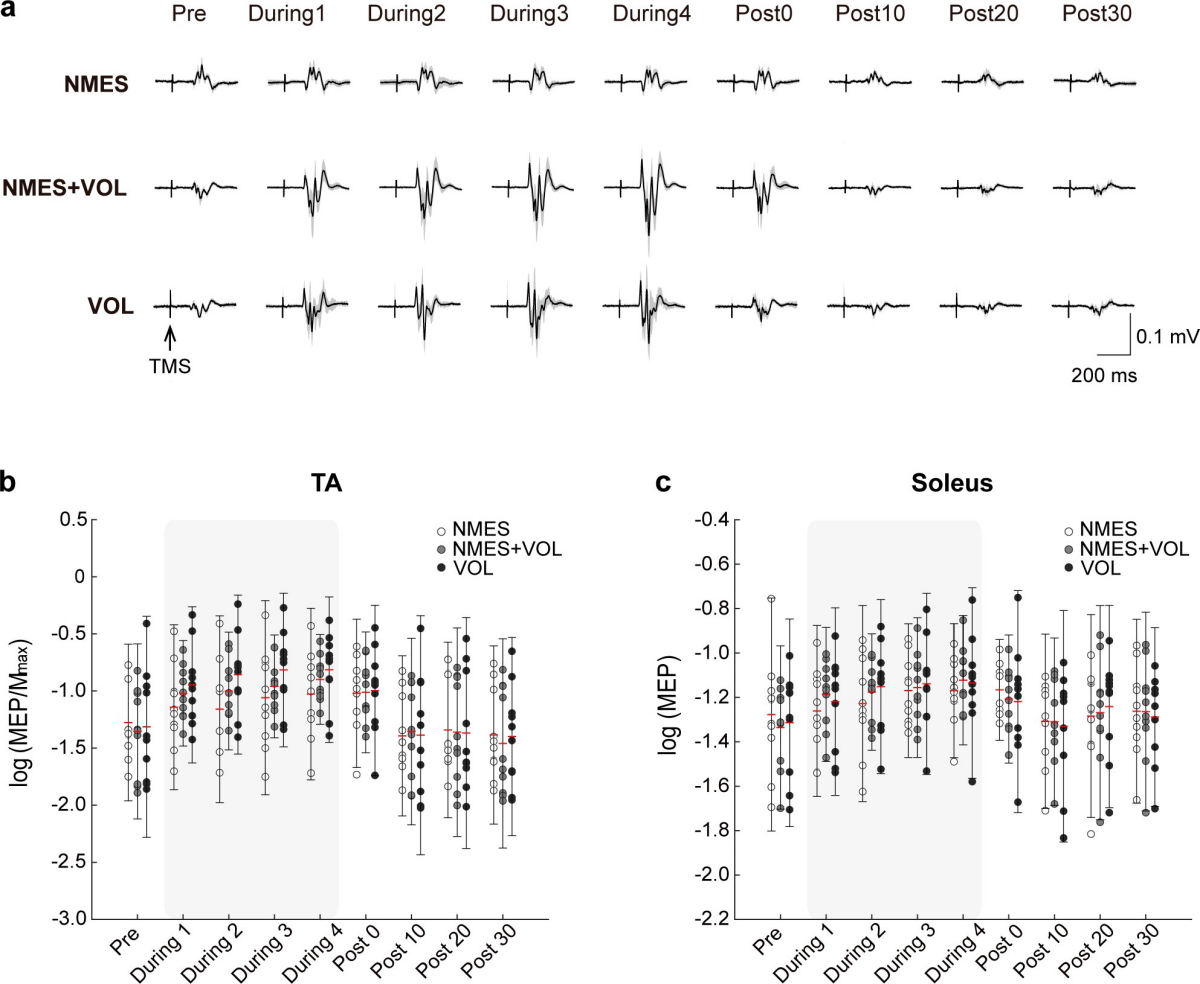
**(a)** MEPs in the TA recorded before starting the interventions (Pre), during the interventions (During1–4), and after the interventions (Post0–30) from a representative participant. Traces indicate average MEP responses; **(b)** Changes in the TA MEP/M_max_; **(c)** Changes in the soleus MEP. Circles represent each individual data and error bars represent 2 standard deviation. The red lines indicate mean for each time point. Abbreviations: MEP, motor evoked potential; TA, tibialis anterior.

Representative MEP traces of the TA muscle for three intervention sessions are shown in Fig 3A. The TA MEP/M_max_ results are shown in Fig 3B. There were significant main effects of intervention (*F*_2,207_ *=* 11.43, *p* < .001, partial η^2^ = .099) and time (*F*_7,204_ *=* 35.94, *p* < .001, partial η^2^ = .552) factors, but no significant interaction (*F*_14,204_ *=* 1.28, *p* = .223, partial η^2^ = .081). Post hoc pairwise comparisons revealed that TA MEP/M_max_ was significantly higher in the NMES+VOL (mean difference: 0.11, 95% confidence interval (CI): 0.03, 0.18; *p* = .002), and VOL (mean difference: 0.14, 95% CI: 0.07, 0.22; *p* < .001), compared to the NMES. Dunnett’s test showed that During4 and Post0 were significantly higher compared to Pre time point in the NMES, while from During1 to Post0 were significantly higher compared to Pre time point in the NMES+VOL and VOL (Table 2).

**Table 2:** Results of Dunnett’s test for the TA MEP/M_max_ differences between Pre and other time points for each intervention.

		NMES				NMES+VOL				VOL			
	Time	Mean Difference (I-J)	*P* value	95% CI		Mean Difference (I-J)	*P* value	95% CI		Mean Difference (I-J)	*P* value	95% CI	
TA MEP/M_max_													
(J)	Pre												
(I)	During1	0.13	.54	-0.11	0.38	0.33*	< .001	0.16	0.51	0.32*	< .01	0.08	0.56
	During2	0.12	.69	-0.13	0.36	0.35*	< .001	0.18	0.53	0.44*	< .001	0.20	0.68
	During3	0.22	.10	-0.03	0.46	0.39*	< .001	0.22	0.57	0.48*	< .001	0.25	0.72
	During4	0.25*	.04	0.01	0.49	0.46*	< .001	0.28	0.63	0.50*	< .001	0.26	0.73
	Post0	0.26*	.04	0.01	0.5	0.34*	< .001	0.17	0.52	0.34*	< .01	0.10	0.58
	Post10	-0.12	.68	-0.36	0.13	0	>.99	-0.17	0.17	0.00	.92	-0.24	0.24
	Post20	-0.07	.98	-0.31	0.18	-0.01	>.99	-0.18	0.17	-0.04	.98	-0.28	0.20
	Post30	-0.11	.75	-0.35	0.13	-0.1	.45	-0.28	0.07	-0.10	.84	-0.34	0.14
Soleus MEP													
(J)	Pre												
(I)	During1	0.02	>.99	-0.12	0.15	0.15*	< .01	0.05	0.25	0.1	.09	-0.01	0.2
	During2	0.05	.90	-0.09	0.19	0.16*	< .001	0.06	0.26	0.16*	< .001	0.06	0.27
	During3	0.11	.19	-0.03	0.25	0.18*	< .001	0.08	0.28	0.18*	< .001	0.07	0.28
	During4	0.11	.19	-0.03	0.25	0.21*	< .001	0.12	0.31	0.18*	< .001	0.07	0.28
	Post0	0.11	.17	-0.03	0.25	0.13*	< .01	0.03	0.23	0.1	.09	-0.01	0.2
	Post10	-0.03	.99	-0.17	0.11	0.03	.98	-0.07	0.12	-0.02	>.99	-0.12	0.09
	Post20	-0.01	>.99	-0.14	0.13	0.07	.31	-0.03	0.16	0.07	.29	-0.03	0.18
	Post30	0.02	>.99	-0.12	0.15	0.07	.25	-0.03	0.17	0.03	.98	-0.08	0.13
Error													
(J)	Pre												
(I)	Post0	-0.02	.99	-0.21	0.16	-0.07	.92	-0.36	0.21	-0.05	.93	-0.24	0.15
	Post10	0.01	>.99	-0.17	0.19	-0.03	.99	-0.31	0.24	-0.09	.51	-0.28	0.09
	Post20	0	>.99	-0.18	0.19	-0.25	.09	-0.53	0.03	-0.02	.99	-0.21	0.16
	Post30	0.03	.98	-0.15	0.21	-0.05	.97	-0.33	0.23	-0.07	.69	-0.26	0.11
Reaction time													
(J)	Pre												
(I)	Post0	0	>.99	-0.02	0.03	0	>.99	-0.03	0.02	0.02	.87	-0.04	0.08
	Post10	-0.01	.79	-0.03	0.02	0	>.99	-0.02	0.03	0.05	.18	-0.01	0.11
	Post20	0	>.99	-0.02	0.02	0.01	.82	-0.02	0.03	0.05	.16	-0.01	0.11
	Post30	-0.01	.77	-0.03	0.02	0.01	.78	-0.02	0.03	0.02	.77	-0.04	0.08

* *p* < .05

Abbreviations: CI, confidence interval; MEP, motor evoked potential; NMES, neuromuscular electrical stimulation; TA, tibialis anterior; VOL, voluntary contractions.

### Soleus (non-targeted muscle) MEP

The soleus MEP results are shown in Fig 3C. There were significant main effects of intervention (*F*_2,210_ *=* 5.56, *p* = .004, partial η^2^ = .050) and time (*F*_7,206_ *=* 9.78, *p* < .001, partial η^2^ = .249) factors, but no significant interaction (*F*_14,206_ *=* .57, *p* = .890, partial η^2^ = .977). Post hoc pairwise comparisons revealed that soleus MEP was significantly higher in NMES+VOL compared to NMES (mean difference: 0.06, 95% CI: 0.02, 0.10; *p* = .004). Dunnett’s test showed that from During1 to Post0 and from During2 to During4 were significantly higher compared to Pre time point in the NMES+VOL and VOL, respectively (Table 1).

### M_max_

**Fig 4:**
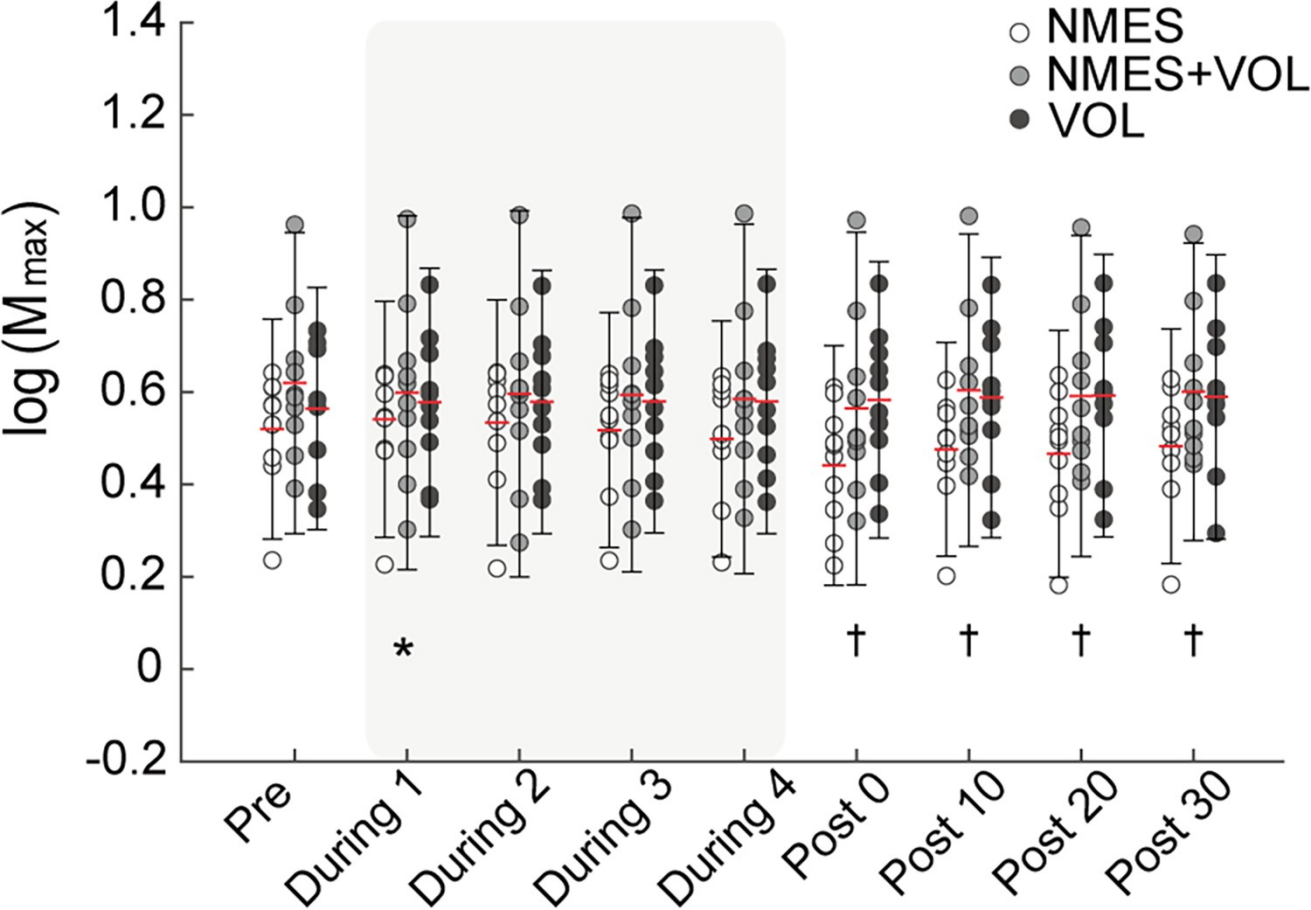
Changes in the M_max_. Circles represent each individual data and error bars represent 2 standard deviation. The red lines indicate mean for each time point. Legend: *p < .05 indicates significant differences between NMES and NMES+VOL. †*p* < .05 indicates significant differences between NMES and VOL. Abbreviations: NMES, neuromuscular electrical stimulation; VOL, voluntary contractions.

The M_max_ results are shown in Fig 4. There were significant main effects of intervention (*F*_2,210_ *=* 24.01, *p* < .001, partial η^2^ = .186) and time (*F*_7,205_ *=* 2.30, *p* = .028, partial η^2^ = .0728) factors, as well as significant interaction (*F*_14,205_ *=* 1.81, *p* = .039, partial η^2^ = .110) for the M_max_. Post-hoc pairwise comparisons between interventions at each time point showed that M_max_ was significantly lower during NMES+VOL compared to NMES at During1 (mean difference: -0.05, 95% CI: -0.10, -0.001; *p* = .042) and the M_max_ was significantly lower during NMES compared to VOL from Post0 to Post30 (all *p* < .05).

### Motor control performance

**Fig 5:**
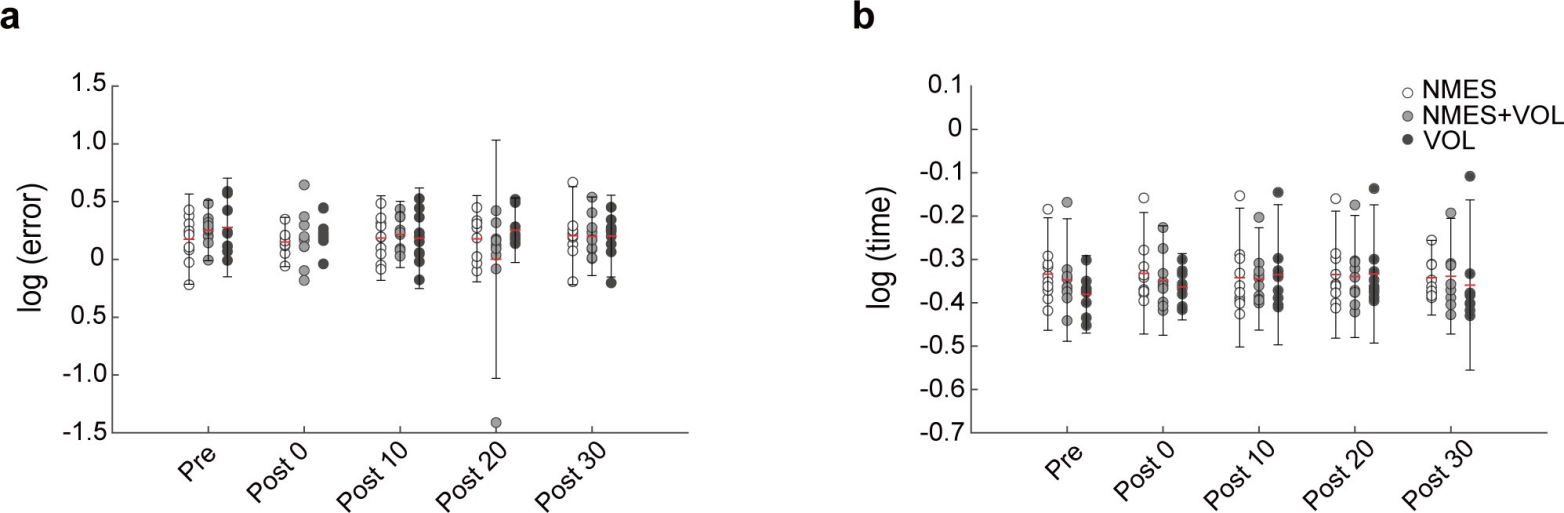
Changes in the motor control of ankle dorsiflexion **(a)** error; and **(b)** reaction time during force-matching task. Circles represent each individual data and error bars represent 2 standard deviation. The red lines indicate mean for each time point. Abbreviations: NMES, neuromuscular electrical stimulation; VOL, voluntary contractions.

The force-matching task results are shown in Fig 5. There were no main effects of intervention (*F*_2,97_ *=* .95, *p* = .389, partial η^2^ = .019) and time (*F*_3,96_ *=* .47, *p* = .707, partial η^2^ = .014) factors, as well as no significant interaction (*F*_6,96_ *=* 24.01, *p* = .388, partial η^2^ = .063) for the error. There were no main effects of intervention (*F*_2,93_ *=* .08, *p* = .920, partial η^2^ = .002) and time (*F*_3,92_ *=* .82, *p* = .488, partial η^2^ = .026) factors, as well as no significant interaction (*F*_6,92_ *=* .98, *p* = .443, partial η^2^ = .060) for the reaction time. Dunnett’s test showed that there were no significant differences between Pre and other time points on both error and reaction time in all interventions (Table 1).

## Discussion

Our current study explored whether superior combined effects would exist when NMES and voluntary ankle dorsiflexion are combined by comparing corticospinal excitability and motor control responses after the interventions specifically designed to produce matched force outputs. Consequently, although the combined intervention involving NMES and voluntary drive (i.e., NMES+VOL) showed similar effects on corticospinal excitability facilitation as when voluntary contractions were performed alone, greater effects were observed compared to NMES alone. Regarding the time course change, during interventions which required voluntary contractions (i.e., NMES+VOL and VOL), corticospinal excitability was facilitated immediately after starting the intervention, but it returned to Pre level within 10 min after interventions were over. Motor control was not affected by any of the interventions.

### Combined intervention effects

Our results showed that corticospinal excitability (MEP/M_max_) of the activated TA muscle was increased to a larger extent during the combined intervention compared to when NMES was applied alone. However, contrary to our hypothesis, the superior effects were not observed compared to the voluntary contractions. Previously, Khaslavskaia and Sinkjaer et al. [5] demonstrated greater corticospinal facilitation after the combined intervention of NMES and voluntary muscle contractions compared to when these were applied alone. However, they simply combined the two interventions, which resulted in a larger total force output compared to each single intervention. Similarly, Jochumsen et al. [7] also showed significantly increased corticospinal excitability when NMES was applied together with voluntary contractions, but the produced force in the combined intervention was also larger than that in each single intervention. The larger force is generated through both greater recruitment of motor units by efferent central commands and stronger afferent volleys produced by NMES [52]. Therefore, the greater corticospinal excitability can likely be attributed to larger total force output (i.e., additive effects). On the other hand, a study by Taylor et al. [53] investigated corticospinal excitability modulation under controlled total force output between interventions. They compared corticospinal excitability modulation during voluntary contraction alone and EMG-triggered NMES when participants performed wrist extensor muscle contractions to produce 15% MVC force in both interventions. Consistent with our results, they showed that there were no superior effects of the combined intervention compared to voluntary contraction alone when force output was matched. Taken together, the combined effects shown previously [5, 7] are likely associated with the greater force output in the combined interventions.

It is important to note that the extent of corticospinal excitability might relate to not only the generated total force output but also the neuromodulation effects of the combined intervention (i.e., pairing effects). In this study, voluntarily produced force and NMES generated force output (i.e., 10% MVC force each) during the combined intervention were just half of that during each single intervention (i.e., 20% MVC force). Despite having the same total force output between interventions and the low-level voluntary contractions and NMES, corticospinal excitability during the combined intervention was facilitated to a similar extent to that during voluntary muscle contractions and more than that during NMES alone. Furthermore, the combined intervention resulted in increased corticospinal excitability 4 min after the intervention started, suggesting that even such a low-level combined approach can effectively and rapidly facilitate the motor pathways. A possible mechanism can be explained by the strengthening of pre- and postsynaptic neurons in the spinal cord [17, 19]. When the muscle contractions are generated during NMES, voluntary commands activate motoneurons, while the evoked afferent sensory volleys generated by NMES and somatosensory inputs during voluntary muscle contractions lead to potentiated neurotransmitter release from Ia afferents to motoneurons in the spinal cord [35, 52, 54]. Consequently, simultaneous activation of pre- and postsynaptic terminals may generate neuromodulation effects by strengthening the connections between these terminals [19]. Another explanation is that prolonged elevation of the intrinsic excitability of motoneurons might be induced by repeated voluntary muscle contractions and NMES. Gorassini et al. [55] demonstrated that muscle vibration during voluntary contractions induced prolonged motor unit firing. In addition, it was also previously shown that repeated voluntary muscle contractions induced a decrease in the motor unit recruitment threshold, suggesting enhancement of intrinsic excitability of motoneurons [56]. Thus, our result may be affected by repeated voluntary contractions induced synaptic excitation of motoneurons and NMES which additionally activated motoneurons with prolonged motor unit firing like peripheral afferent inputs (i.e., similar to muscle vibration). Consequently, even though the level of voluntary contraction and NMES intensity was relatively low, this combination might effectively lead to the synaptic facilitation in the spinal cord. Furthermore, it was also demonstrated that peripheral nerve stimulation increased MEPs elicited in the stimulated hand muscle accompanied by unaltered responses in F-wave responses, which suggests that corticospinal excitability likely originates within the motor cortex [24]. Although speculative, there is a possibility that afferent inputs by NMES induced synaptic strengthening in the motor cortex, which resulted in the facilitation of the postsynaptic neurons [18]. In addition, neuroimaging studies have shown that the combination of NEMS and voluntary movements induced greater activation in the somatosensory areas, which are responsible for sensory information processing and sensorimotor integration, compared to voluntary movements alone [38–40]. Specifically, Gandolla et al. [40] suggest that concurrent voluntary movements during NMES increase connections between the primary motor cortex and the primary somatosensory cortex. Given these previous findings, despite the low-level voluntary contractions, excitatory modulation seemed to be induced in the subcortical and/or cortical neural circuits, which supports evidence of facilitatory effects of voluntary engagement during NMES [2, 14–16]. Therefore, our findings imply that voluntary engagement could improve the effects of NMES even through weak muscle contractions. This pairing effect is especially important for individuals who cannot produce sufficient voluntary contractions due to neurological impairments. Our result confirms the fundamental underlying mechanism of NMES to produce purposeful movements in rehabilitation, i.e., functional electrical stimulation (FES) therapy [1, 16, 30] and extends our knowledge related to brain-computer interface-controlled FES technologies [57, 58].

Our results showed that the combined intervention also facilitated MEP responses of the soleus muscle (i.e., antagonist non-activated muscle) to a larger extent compared to the NMES alone intervention. Previous studies have shown that MEPs elicited in the soleus muscle do not reduce but get facilitated during voluntary contraction of the dorsiflexors [59, 60]. It was also demonstrated that there was no significant intracortical inhibition in the soleus muscle when it acted as an antagonist during voluntary ankle dorsiflexion [61]. These findings indicate that the soleus muscle is not inhibitory but facilitatory controlled in the primary motor cortex during dorsiflexion. Therefore, NMES might additionally facilitate cortical circuits involving not only the agonists but also antagonist muscles during voluntary ankle dorsiflexion. Such global effects are in line with those of previous studies that demonstrated the corticospinal [4, 62] and spinal [13] level effects. Taken together, our results suggest that the combined intervention can rapidly elicit global corticospinal excitability facilitation in the lower-limb motor circuits.

### Lack of aftereffects

Notably, in contrast to our hypothesis and previous reports which showed long-lasting neural facilitation after applications of NMES [5, 7–9], our current study showed that corticospinal excitability returned to Pre immediately after all interventions were over. Meanwhile, M_max_ was lower after the NMES intervention compared to voluntary contractions. In accordance with the present result, a previous study also demonstrated a decline of the M-wave amplitude after tetanic stimulation [63], suggesting the peripheral failure of neuromuscular transmission and/or a decrease of the muscle fiber conduction velocity. These peripheral changes might affect a decline in the responses to TMS (i.e., decreased MEP amplitude) and result in the lack of aftereffects. Since the M_max_ response represents the total activation of motor neuron pool and changes when muscle fatigue is present [64], it is likely that muscle fatigue may have occurred during NMES in our study. Although we used a 25% stimulation duty cycle (5 s ON / 19 s OFF) to provide adequate rest (i.e., OFF) intervals, there is still a possibility that muscle fatigue may be responsible for the lack of aftereffects. Previous studies have demonstrated effective approaches to reduce muscle fatigue, such as using multiple electrodes [65, 66], nerve stimulation [66], or modifying the pulse width and stimulation frequency (see [67] for a review). Therefore, future studies should consider alternative NMES protocols to minimize fatigue effects.

There are also other explanations for the lack of aftereffects. First, a rebound phenomenon (i.e., homeostatic regulation) might have caused a depression of the neural excitation after the interventions. It has been suggested that homeostatic control plays an important role to avoid hyper- or hypo-activity of the neural circuits [68]. Since the interventions required voluntary contractions modulated corticospinal activation immediately after the start of interventions, it is speculated that homeostatic regulation might be engaged in stabilizing neural activation. Second, relatively low muscle contractions and a short duration of NMES might be insufficient to lead to long-lasting corticospinal facilitation. Our study used a total of 20% of MVC force for 16 min to prevent muscle fatigue and discomfort, whereas previous studies which showed significant corticospinal aftereffects applied 30% of MVC force for 30 min [5] or longer NMES intervention [8, 9]. In order to induce neuroplasticity, a certain amount of synaptic activity is required [69]. Thus, longer interventions may be required to facilitate corticospinal neuroplasticity after the intervention.

### Motor control performance

Our results showed that corticospinal excitability was modulated until immediately after the interventions (i.e., at Post0), and that the combined intervention facilitated corticospinal excitability to a greater extent than NMES alone. However, motor control was not affected after any interventions. These results suggest that even though corticospinal excitability was facilitated, motor control performance might be unchanged by short-term NMES application. It is worth noting that these results may be attributed to the motor task. Previous studies showed that preconditioning NMES for acquiring skilled-motor tasks induced facilitation of cortical excitability along with rapid improvements in fine motor control [33] as well as visuomotor adaptation [34] in healthy participants. On the other hand, the task in our current study seems to be relatively simple for healthy participants and their performance levels might be high enough at the Pre time point such that participants reached a plateau. Therefore, corticospinal excitability facilitation may affect motor control performance during more complex tasks. Furthermore, contrary to our current study findings, it has previously been shown that EMG-triggered NMES for 2 to 10 weeks induced improvement of motor control reaction time [41, 70] and accuracy [41] in stroke patients. Therefore, longer duration combined interventions might promote motor control improvements in neurological impairment populations.

### Limitations

The small sample size is one of the limitations in this study. Thus, further investigation with more participants is needed to generalize our findings to a large population. It should be noted that participants were not asked to abstain from caffeine consumption before the experiments. Since it is known that caffeine intake can increase corticospinal excitability [71], it cannot be ruled out that it may have had a confounding effect in our current study. This point must be controlled in future studies. In addition, during voluntary muscle contractions of the TA muscle, participants’ legs were not secured. Although the level of muscle contractions was relatively low, it cannot be ruled out that contractions of knee extensors may have affected MEP responses of the TA muscle. Furthermore, as mentioned before, muscle fatigue is a crucial issue using NMES. Developing NMES applications which induce less muscle fatigue would be more effective to lead to the combined effects. Nevertheless, our results herein provide insights into the underlying mechanisms of NMES neuromodulation under the study paradigm for future investigations.

## Conclusions

Our study specifically focused on the combined effects of NMES and voluntary muscle contractions on corticospinal excitability facilitation and motor control, comparing each under matched levels of total force generation during the intervention. Our findings suggest that motor control might not be affected by short-term NMES delivery. Importantly, although superior effects of the combined intervention were not demonstrated compared to voluntary muscle contractions, greater effects on corticospinal excitability facilitation during the intervention compared to NMES alone were shown. This result suggests that voluntary engagement could improve the effects of NMES, even when the voluntary contractions are not exceedingly strong. This is especially important for individuals with neurological impairments who are unable to generate sufficient voluntary motor outputs.

## Supporting information

S1 Dataset Raw data. (XLSX)

## References

[1] Popovic MR, Curt A, Keller T, Dietz V. Functional electrical stimulation for grasping and walking: Indications and limitations. Spinal Cord. 2001;39: 403–412. doi: 10.1038/sj.sc.3101191 11512070

[2] Gandolla M, Ward NS, Molteni F, Guanziroli E, Ferrigno G, Pedrocchi A. The Neural Correlates of Long-Term Carryover following Functional Electrical Stimulation for Stroke. Neural Plast. 2016;2016: 1–13. doi: 10.1155/2016/4192718 27073701 PMC4814690

[3] Street T, Singleton C. A clinically meaningful training effect in walking speed using functional electrical stimulation for motor-incomplete spinal cord injury. J Spinal Cord Med. 2018;41: 361–366. doi: 10.1080/10790268.2017.1392106 29108487 PMC6055946

[4] Thompson AK, Stein RB. Short-term effects of functional electrical stimulation on motor-evoked potentials in ankle flexor and extensor muscles. Exp Brain Res. 2004;159: 491–500. doi: 10.1007/s00221-004-1972-4 15243732

[5] Khaslavskaia S, Sinkjaer T. Motor cortex excitability following repetitive electrical stimulation of the common peroneal nerve depends on the voluntary drive. Exp Brain Res. 2005;162: 497–502. doi: 10.1007/s00221-004-2153-1 15702321

[6] Mang CS, Bergquist AJ, Roshko SM, Collins DF. Loss of short-latency afferent inhibition and emergence of afferent facilitation following neuromuscular electrical stimulation. Neurosci Lett. 2012;529: 80–85. doi: 10.1016/j.neulet.2012.08.072 22985510

[7] Jochumsen M, Niazi IK, Signal N, Nedergaard RW, Holt K, Haavik H, et al. Pairing Voluntary Movement and Muscle-Located Electrical Stimulation Increases Cortical Excitability. Front Hum Neurosci. 2016;10: 1–8. doi: 10.3389/fnhum.2016.00482 27733823 PMC5039207

[8] Knash ME, Kido A, Gorassini M, Chan KM, Stein RB. Electrical stimulation of the human common peroneal nerve elicits lasting facilitation of cortical motor-evoked potentials. Exp Brain Res. 2003;153: 366–377. doi: 10.1007/s00221-003-1628-9 14610631

[9] Thompson AK, Lapallo B, Duffield M, Abel BM, Pomerantz F. Repetitive common peroneal nerve stimulation increases ankle dorsiflexor motor evoked potentials in incomplete spinal cord lesions. Exp Brain Res. 2011;210: 143–152. doi: 10.1007/s00221-011-2607-1 21360230

[10] Dobkin BH. Motor rehabilitation after stroke, traumatic brain, and spinal cord injury: common denominators within recent clinical trials. Curr Opin Neurol. 2009;22: 563–569. doi: 10.1097/WCO.0b013e3283314b11 19724226 PMC4077333

[11] Kato T, Sasaki A, Yokoyama H, Milosevic M, Nakazawa K. Effects of neuromuscular electrical stimulation and voluntary commands on the spinal reflex excitability of remote limb muscles. Exp Brain Res. 2019;237: 3195–3205. doi: 10.1007/s00221-019-05660-6 31602493 PMC6882749

[12] Takahashi Y, Kawakami M, Yamaguchi T, Idogawa Y, Tanabe S, Kondo K, et al. Effects of leg motor imagery combined with electrical stimulation on plasticity of corticospinal excitability and spinal reciprocal inhibition. Front Neurosci. 2019;13: 1–9. doi: 10.3389/fnins.2019.00149 30846928 PMC6393385

[13] Milosevic M, Masugi Y, Obata H, Sasaki A, Popovic MR, Nakazawa K. Short-term inhibition of spinal reflexes in multiple lower limb muscles after neuromuscular electrical stimulation of ankle plantar flexors. Exp Brain Res. 2019;237: 467–476. doi: 10.1007/s00221-018-5437-6 30460394

[14] Barsi GI, Popovic DB, Tarkka IM, Sinkjær T, Grey MJ. Cortical excitability changes following grasping exercise augmented with electrical stimulation. Exp Brain Res. 2008;191: 57–66. doi: 10.1007/s00221-008-1495-5 18663439

[15] Hara Y, Obayashi S, Tsujiuchi K, Muraoka Y. The effects of electromyography-controlled functional electrical stimulation on upper extremity function and cortical perfusion in stroke patients. Clin Neurophysiol. 2013;124: 2008–2015. doi: 10.1016/j.clinph.2013.03.030 23706813

[16] Everaert DG, Thompson AK, Chong SL, Stein RB. Does Functional Electrical Stimulation for Foot Drop Strengthen Corticospinal Connections? Neurorehabil Neural Repair. 2009;24: 168–177. doi: 10.1177/1545968309349939 19861590

[17] Hebb DO. the Organization of Behavior. J Appl Behav Anal. 1949;25: 575–577. doi: 10.1901/jaba.1992.25–575

[18] Knutson JS, Fu MJ, Sheffler LR, Chae J. Neuromuscular Electrical Stimulation for Motor Restoration in Hemiplegia. Phys Med Rehabil Clin N Am. 2016;26: 729–745. doi: 110.1016/j.bbi.2017.04.008 10.1016/j.pmr.2015.06.002 PMC4630679 26522909

[19] Rushton DN. Functional Electrical Stimulation and rehabilitation—an hypothesis. Med Eng Phys. 2003;52: 507–513. doi: 10.1016/s1350-4533(02)00040-1 12485788

[20] Chipchase LS, Schabrun SM, Hodges PW. Corticospinal excitability is dependent on the parameters of peripheral electric stimulation: A preliminary study. Arch Phys Med Rehabil. 2011;92: 1423–1430. doi: 10.1016/j.apmr.2011.01.011 21620374

[21] Andrews RK, Schabrun SM, Ridding MC, Galea MP, Hodges PW, Chipchase LS. The effect of electrical stimulation on corticospinal excitability is dependent on application duration: A same subject pre-post test design. J Neuroeng Rehabil. 2013;10: 51. doi: 10.1186/1743-0003-10-51 23758902 PMC3688368

[22] Perez MA, Lungholt BKS, Nyborg K, Nielsen JB. Motor skill training induces changes in the excitability of the leg cortical area in healthy humans. Exp Brain Res. 2004;159: 197–205. doi: 10.1007/s00221-004-1947-5 15549279

[23] Lotze M, Braun C, Birbaumer N, Anders S, Cohen LG. Motor learning elicited by voluntary drive. Brain. 2003;126: 866–872. doi: 10.1093/brain/awg079 12615644

[24] Ridding MC, Brouwer B, Miles TS, Pitcher JB, Thompson PD. Changes in muscle responses to stimulation of the motor cortex induced by peripheral nerve stimulation in human subjects. Exp brain Res. 2000;131: 135–43. doi: 10.1007/s002219900269 10759179

[25] Golaszewski SM, Bergmann J, Christova M, Kunz AB, Kronbichler M, Rafolt D, et al. Modulation of motor cortex excitability by different levels of whole-hand afferent electrical stimulation. Clin Neurophysiol. 2012;123: 193–199. doi: 10.1016/j.clinph.2011.06.010 21764634

[26] McKay D, Brooker R, Giacomin P, Ridding M, Miles T. Time course of induction of increased human motor cortex excitability by nerve stimulation. Neuroreport. 2002;13: 1271–1273. doi: 10.1097/00001756-200207190-00011 12151785

[27] Collins DF. Central contributions to contractions evoked by tetanic neuromuscular electrical stimulation. Exerc Sport Sci Rev. 2007;35: 102–109. doi: 10.1097/jes.0b013e3180a0321b 17620928

[28] Chipchase LS, Schabrun SM, Hodges PW. Peripheral electrical stimulation to induce cortical plasticity: A systematic review of stimulus parameters. Clinical Neurophysiology. 2011;122: 456–463. doi: 10.1016/j.clinph.2010.07.025 20739217

[29] Saito K, Yamaguchi T, Yoshida N, Tanabe S, Kondo K, Sugawara K. Combined effect of motor imagery and peripheral nerve electrical stimulation on the motor cortex. Exp Brain Res. 2013;227: 333–342. doi: 10.1007/s00221-013-3513-5 23591692

[30] Marquez-Chin C, Popovic MR. Functional electrical stimulation therapy for restoration of motor function after spinal cord injury and stroke: A review. Biomed Eng Online. 2020;19: 1–25. doi: 10.1186/s12938-020-00773-4 32448143 PMC7245767

[31] Muellbacher W, Ziemann U, Boroojerdi B, Cohen L, Hallett M. Role of the human motor cortex in rapid motor learning. Exp Brain Res. 2001;136: 431–438. doi: 10.1007/s002210000614 11291723

[32] Ljubisavljevic M. Transcranial magnetic stimulation and the motor learning-associated cortical plasticity. Exp Brain Res. 2006;173: 215–222. doi: 10.1007/s00221-006-0538-z 16733699

[33] McDonnell MN, Ridding MC. Afferent stimulation facilitates performance on a novel motor task. Exp Brain Res. 2006;170: 109–115. doi: 10.1007/s00221-005-0192-x 16328288

[34] Summers SJ, Schabrun SM, Marinovic W, Chipchase LS. Peripheral electrical stimulation increases corticomotor excitability and enhances the rate of visuomotor adaptation. Behav Brain Res. 2017;322: 42–50. doi: 10.1016/j.bbr.2017.01.016 28089855

[35] Collins DF, Burke D, Gandevia SC. Sustained contractions produced by plateau-like behaviour in human motoneurones. J Physiol. 2002;538: 289–301. doi: 10.1113/jphysiol.2001.012825 11773336 PMC2290016

[36] Dean JC, Yates LM, Collins DF. Turning off the central contribution to contractions evoked by neuromuscular electrical stimulation. Muscle and Nerve. 2008;38: 978–986. doi: 10.1002/mus.21007 18537146

[37] Enoka RM, Duchateau J. Rate Coding and the Control of Muscle Force. Cold Spring Harb Perspect Med. 2017;7: a029702. doi: 10.1101/cshperspect.a029702 28348173 PMC5629984

[38] Christensen MS, Grey MJ. Modulation of proprioceptive feedback during functional electrical stimulation: an fMRI study. Eur J Neurosci. 2013;37: 1766–1778. doi: 10.1111/ejn.12178 23461704

[39] Iftime-Nielsen SD, Christensen MS, Vingborg RJ, Sinkjær T, Roepstorff A, Grey MJ. Interaction of electrical stimulation and voluntary hand movement in SII and the cerebellum during simulated therapeutic functional electrical stimulation in healthy adults. Hum Brain Mapp. 2012;33: 40–49. doi: 10.1002/hbm.21191 21591025 PMC6870182

[40] Gandolla M, Ferrante S, Molteni F, Guanziroli E, Frattini T, Martegani A, et al. Re-thinking the role of motor cortex: Context-sensitive motor outputs? Neuroimage. 2014;91: 366–374. doi: 10.1016/j.neuroimage.2014.01.011 24440530 PMC3988837

[41] Shin HK, Cho SH, Jeon H seon, Lee YH, Song JC, Jang SH, et al. Cortical effect and functional recovery by the electromyography-triggered neuromuscular stimulation in chronic stroke patients. Neurosci Lett. 2008;442: 174–179. doi: 10.1016/j.neulet.2008.07.026 18644424

[42] Schneiders AG, Sullivan SJ, Malley KJO, Clarke S V, Knappstein SA, Taylor LJ. A Valid and Reliable Clinical Determination of Footedness. Phys Med Rehabil. 2010;2: 835–841. doi: 10.1016/j.pmrj.2010.06.004 20869683

[43] Rossini PM, Burke D, Chen R, Cohen LG, Daskalakis Z, Iorio R Di, et al. Non-invasive electrical and magnetic stimulation of the brain, spinal cord, roots and peripheral nerves: Basic principles and procedures for routine clinical and research application. An updated report from an I.F.C.N. Committee. Clin Neurophysiol. 2015;126: 1071–1107. doi: 10.1016/j.clinph.2015.02.001 25797650 PMC6350257

[44] Yamaguchi A, Milosevic M, Sasaki A, Nakazawa K. Force Control of Ankle Dorsiflexors in Young Adults: Effects of Bilateral Control and Leg Dominance. J Mot Behav. 2020;52: 226–235. doi: 10.1080/00222895.2019.1609408 31084418

[45] Yamaguchi A, Sasaki A, Masugi Y, Milosevic M, Nakazawa K. Changes in corticospinal excitability during bilateral and unilateral lower-limb force control tasks. Exp Brain Res. 2020;238: 1977–1987. doi: 10.1007/s00221-020-05857-0 32591958

[46] Gueugneau N, Grosprêtre S, Stapley P, Lepers R. High-frequency neuromuscular electrical stimulation modulates interhemispheric inhibition in healthy humans. J Neurophysiol. 2016;117: 467–475. doi: 10.1152/jn.00355.2016 27832594 PMC5263217

[47] Terao Y, Ugawa Y, Hanajima R, Machii K, Furubayashi T, Mochizuki H, et al. Predominant activation of II-waves from the leg motor area by transcranial magnetic stimulation. Brain Res. 2000;859: 137–146. doi: 10.1016/S0006-8993(00)01975-2 10720623

[48] Roy FD, Norton JA, Gorassini MA. Role of sustained excitability of the leg motor cortex after transcranial magnetic stimulation in associative plasticity. J Neurophysiol. 2007;98: 657–667. doi: 10.1152/jn.00197.2007 17537908

[49] Veldman MP, Zijdewind I, Solnik S, Maffiuletti NA, Berghuis KMM, Javet M, et al. Direct and crossed effects of somatosensory electrical stimulation on motor learning and neuronal plasticity in humans. Eur J Appl Physiol. 2015;115: 2505–2519. doi: 10.1007/s00421-015-3248-z 26335625 PMC4635177

[50] Davranche K, Temesi J, Verges S, Hasbroucq T. Transcranial magnetic stimulation probes the excitability of the primary motor cortex: A framework to account for the facilitating effects of acute whole-body exercise on motor processes. J Sport Heal Sci. 2015;4: 24–29. doi: 10.1016/j.jshs.2014.09.001

[51] Baayen RH, Davidson DJ, Bates DM. Mixed-effects modeling with crossed random effects for subjects and items. J Mem Lang. 2008;59: 390–412. doi: 10.1016/j.jml.2007.12.005

[52] Bergquist AJ, Clair JM, Lagerquist O, Mang CS, Okuma Y, Collins DF. Neuromuscular electrical stimulation: Implications of the electrically evoked sensory volley. Eur J Appl Physiol. 2011;111: 2409–2426. doi: 10.1007/s00421-011-2087-9 21805156

[53] Taylor L, Lewis GN, Taylor D. Short-term effects of electrical stimulation and voluntary activity on corticomotor excitability in healthy individuals and people with stroke. J Clin Neurophysiol. 2012;29: 237–243. doi: 10.1097/WNP.0b013e3182570f17 22659717

[54] Collins DF, Burke D, Gandevia SC. Large involuntary forces consistent with plateau-like behavior of human motoneurons. J Neurosci. 2001;21: 4059–4065. doi: 10.1523/JNEUROSCI.21-11-04059.2001 11356893 PMC6762712

[55] Gorassini MA, Bennett DJ, Yang JF. Self-sustained firing of human motor units. Neurosci Lett. 1998;247: 13–16. doi: 10.1016/s0304-3940(98)00277-8 9637398

[56] Gorassini M, Yang JF, Siu M, Bennett DJ. Intrinsic activation of human motoneurons: Reduction of motor unit recruitment thresholds by repeated contractions. J Neurophysiol. 2002;87: 1859–1866. doi: 10.1152/jn.00025.2001 11929907

[57] Milosevic M, Marquez-Chin C, Masani K, Hirata M, Nomura T, Popovic MR, et al. Why brain-controlled neuroprosthetics matter: mechanisms underlying electrical stimulation of muscles and nerves in rehabilitation. Biomed Eng Online. 2020;19: 1–30. doi: 10.1186/s12938-020-00824-w 33148270 PMC7641791

[58] Jovanovic LI, Kapadia N, Zivanovic V, Rademeyer HJ, Alavinia M, McGillivray C, et al. Brain–computer interface-triggered functional electrical stimulation therapy for rehabilitation of reaching and grasping after spinal cord injury: a feasibility study. Spinal Cord Ser Cases. 2021;7: 24. doi: 10.1038/s41394-020-00380-4 33741900 PMC7979732

[59] Valls-Solé J, Alvarez R, Tolosa ES. Responses of the soleus muscle to transcranial magnetic stimulation. Electroencephalogr Clin Neurophysiol Evoked Potentials. 1994;93: 421–427. doi: 10.1016/0168-5597(94)90148-1 7529691

[60] Goulart F, Valls-Solé J. Reciprocal changes of excitability between tibialis anterior and soleus during the sit-to-stand movement. Exp Brain Res. 2001;139: 391–397. doi: 10.1007/s002210100771 11534862

[61] Lauber B, Gollhofer A, Taube W. Differences in motor cortical control of the soleus and tibialis anterior. J Exp Biol. 2018;221: jeb174680. doi: 10.1242/jeb.174680 30194250

[62] Mang CS, Clair JM, Collins DF. Neuromuscular electrical stimulation has a global effect on corticospinal excitability for leg muscles and a focused effect for hand muscles. Exp Brain Res. 2011;209: 355–363. doi: 10.1007/s00221-011-2556-8 21286692

[63] Cupido CM, Galea V, McComas AJ. Potentiation and depression of the M wave in human biceps brachii. J Physiol. 1996;491: 541–550. doi: 10.1113/jphysiol.1996.sp021238 8866877 PMC1158748

[64] Crone C, Johnsen LL, Hultborn H, Ørsnes GB. Amplitude of the maximum motor response (M(max)) in human muscles typically decreases during the course of an experiment. Exp Brain Res. 1999;124: 265–270. doi: 10.1007/s002210050621 9928849

[65] Bergquist AJ, Babbar V, Ali S, Popovic MR, Masani K. Fatigue reduction during aggregated and distributed sequential stimulation. Muscle and Nerve. 2017;56: 271–281. doi: 10.1002/mus.25465 27862023

[66] Buckmire A, Arakeri T, Reinhard JP, Fuglevand AJ. Mitigation of excessive fatigue associated with functional electrical stimulation. J Neural Eng. 2018;15: 1672–1673. doi: 10.1088/1741-2552/aade1c 30168443 PMC6370345

[67] Barss TS, Ainsley EN, Claveria-Gonzalez FC, Luu MJ, Miller DJ, Wiest MJ, et al. Utilizing Physiological Principles of Motor Unit Recruitment to Reduce Fatigability of Electrically-Evoked Contractions: A Narrative Review. Arch Phys Med Rehabil. 2018;99: 779–791. doi: 10.1016/j.apmr.2017.08.478 28935232

[68] Turrigiano GG, Nelson SB. Homeostatic plasticity in the developing nervous system. Nat Rev Neurosci. 2004;5: 97–107. doi: 10.1038/nrn1327 14735113

[69] Reis J, Swayne OB, Vandermeeren Y, Camus M, Dimyan MA, Harris-love M, et al. Contribution of transcranial magnetic stimulation to the understanding of cortical mechanisms involved in motor control. J Physiol. 2008;586: 325–351. doi: 10.1113/jphysiol.2007.144824 17974592 PMC2375593

[70] Cauraugh JH, Kim S. Two coupled motor recovery protocols are better than one: Electromyogram-triggered neuromuscular stimulation and bilateral movements. Stroke. 2002;33: 1589–1594. doi: 10.1161/01.str.0000016926.77114.a6 12052996

[71] Gandevia SC, Taylor JL. Supraspinal fatigue: The effects of caffeine on human muscle performance. J Appl Physiol. 2006;100: 1749–1750. doi: 10.1152/japplphysiol.00121.2006 16714410

